# OP25 Rapid Molecular Tests For Detecting Multidrug-Resistant Microorganisms: An Overview Of Systematic Reviews Within A Living Evidence Project

**DOI:** 10.1017/S0266462324000886

**Published:** 2025-01-07

**Authors:** Valentin Imeshtari, Nora Ibargoyen Roteta, Gaizka Benguria Arrate, Lorea Galnares Cordero, Maria X. Rojas, Iñaki Gutierrez-Ibarluzea

## Abstract

**Introduction:**

Ranked among the top 10 global health threats, antimicrobial resistance (AMR) prompts concern. Embracing a living evidence synthesis (LES), we address this concern by informing health decision-makers about molecular tests for swift detection of multidrug-resistant organisms (MDROs). This updated HTA report for the Spanish National Health System prioritizes decisions based on the most current evidence, adapting to emerging technologies and evidence.

**Methods:**

The LES commenced with a baseline synthesis, shaping the initial HTA report regarding rapid molecular test impact on safety, efficacy, effectiveness, and patient outcomes in suspected infection cases. Based on this, on 12 July 2023, we initiated a 12-month evidence monitoring process. Utilizing the Living Evidence to Inform Health Decisions (LE-IHD) framework and interactive tools, we conducted ongoing baseline synthesis and evidence tracking. Artificial intelligence (AI) and the Living Overview of Evidence (L.OVE) platform aided in continual evidence identification. Tri-monthly scans of trial registries unveiled ongoing studies. New eligible studies were rigorously assessed. Updates in HTA conclusions were in line with this synthesis.

**Results:**

The baseline synthesis identified 25 systematic reviews that suggested that the use of rapid molecular test for the identification of pulmonary tuberculosis showed good performance, but less evidence or low-quality evidence was available for other medical conditions or on patient outcomes. During the conference, we will report on 11 months of monitoring and regular updates, including key messages on changes in the evidence synthesis conclusions whenever there are substantial updates of the HTA report.

**Conclusions:**

The living evidence approach enables timely updates of conclusions for HTA reports relying on low- and very low-quality evidence, enhancing their significance in decision-making. The LE-IHD framework streamlines tasks for HTA developers in planning and executing LES to inform health decisions.

